# Hepatoprotective Effect of *Opuntia robusta* Fruit Biocomponents in a Rat Model of Thioacetamide-Induced Liver Fibrosis

**DOI:** 10.3390/plants11152039

**Published:** 2022-08-04

**Authors:** Nayeli Amalinalli Pulido-Hornedo, Javier Ventura-Juárez, Fidel Guevara-Lara, Herson Antonio González-Ponce, Esperanza Sánchez-Alemán, Manon Buist-Homan, Han Moshage, Ma. Consolación Martínez-Saldaña

**Affiliations:** 1 Basic Sciences Center, Department of Morphology, Universidad Autónoma de Aguascalientes, Aguascalientes 20100, Mexico; 2 Basic Sciences Center, Department of Chemistry, Universidad Autónoma de Aguascalientes, Aguascalientes 20100, Mexico; 3 Instituto Tecnológico de Aguascalientes, Aguascalientes 20256, Mexico; 4 Unidad de Medicina Familiar 8, Instituto Mexicano del Seguro Social (IMSS), Aguascalientes 20180, Mexico; 5 Department of Gastroenterology and Hepatology, University Medical Center of Groningen, University of Groningen, 9713 Groningen, The Netherlands; 6 Department Laboratory Medicine, University Medical Center Groningen, University of Groningen, 9713 Groningen, The Netherlands

**Keywords:** antioxidants, liver fibrosis, biocompounds, functional foods, oxidative stress, liver injury

## Abstract

Liver fibrosis is a chronic disease associated with oxidative stress that has a great impact on the population mortality. Due to their antioxidant capacity, we evaluated the protective effect of *Opuntia robusta* fruit (*Or*) on liver fibrosis. A nutraceutical characterization of *Or* was performed and a model of fibrosis was induced with thioacetamide (TAA) in Wistar rats. Aminotransferases, reduced glutathione (GSH) and histopathology were evaluated. *Or* contained 436.5 ± 57 mg of Betacyanins equivalents/L., 793 mg of catechin equivalents (CAE)/100 g for flavonoids, 1118 mg of gallic acid equivalents (GAE)/100 g for total phenols, 141.14 mg/100 g for vitamin C and 429.9 μg/100 g for vitamin E. The antioxidant capacity of *Or* was: 2.27 mmol of Trolox^®^ equivalents (TE)/L (DPPH), 62.2 ± 5.0 μmol TE/g (ABTS^•+^), 80.2 ± 11.7 μmol TE/g (FRAP), 247.9 ± 15.6 µmol TE/g (AAPH) and 15.0% of H_2_O_2_ elimination. An increase (*p* < 0.05) of aminotransferases and a decrease (*p* < 0.05) of hepatic GSH was observed in the TAA group compared to the control and the concomitant groups. Histopathology showed changes in the normal architecture of the liver treated with TAA compared to the concomitant treatments. *Or* contains bioactive components with antioxidant capacity, which can reduce fibrotic liver damage.

## 1. Introduction

Fibrosis is an inadequate adaptative response to chronic tissue damage. Oxidative stress is a key factor in the development of liver fibrosis, as it damages hepatocytes and promotes the release of proinflammatory mediators [1]. Liver fibrosis is associated with significantly increased rates of morbidity and mortality worldwide and there are currently no adequate therapies to treat its progression [2].

Liver fibrosis is a dynamic process characterized by an imbalance between extracellular matrix secretion and degradation in response to continuous exposure of the liver to noxious agents. Chronic exposure to certain chemical substances generates oxidative stress that induces secretion of soluble inflammatory and fibrogenic mediators, such as TNF-α, TGF-β, IL1, and IL6, eventually causing hepatocellular damage [3]. These cytokines activate different populations of hepatic cells and cause a sustained inflammatory reaction. One of the cell populations that is activated are hepatic macrophages (Kupffer cells). Once activated they cause the activation of quiescent hepatic stellate cells (qHSC) triggering an imbalance of synthesis and degradation of extracellular matrix components (EMC).

Due to the increase in the incidence and prevalence of liver fibrosis and the relative ineffectiveness of currently used treatments, there is a need for new therapeutic strategies to reduce damage, and thus prevent its progression.

Thioacetamide (TAA) is a compound widely used to simulate the damage generated in the pathogenesis of liver fibrosis. TAA increases reactive oxygen species (ROS) formation via its metabolite, thioacetamide sulfdioxide (TASO_2_), which causes severe oxidative stress along with lipid peroxidation and generation of protein carbonyls and DNA adducts [4]. Oxidative stress in the liver causes activation of hepatic stellate cells (HSCs) due to ROS produced during TAA metabolism. ROS provides paracrine activation signals that induce transdifferentiation of HSCs into myofibroblast-like cells causing an imbalance in EMC synthesis and degradation [5], perpetuating the fibrotic process.

Species of the *Opuntia* genus have great cultural and nutritional importance in Mexico. Eighty three of the 189 species that comprise the genus are found in the north-central zone of this country, in the states of Guanajuato, Hidalgo, Jalisco, Querétaro, San Luis Potosí, Zacatecas and Aguascalientes [6]. *Opuntia* species have many benefits, and their widespread distribution has prompted research on the nutritional properties of their fruits, the prickly pears [7,8], which have been proposed as functional foods. Concentrations of various bioactive compounds have been reported before, mainly betacyanins, total phenols and flavonoids in seeds [9], peel [9,10,11], pulp [9,11], and juice [12] of different species of this genus. However, the fruits of each harvest have different concentrations of secondary metabolites since these depend on external environmental conditions and stimuli, such as humidity, temperature, season, altitude, pollution, etc. [9,12,13] Thus, it is important to perform a nutraceutical characterization each time a new study is carried out in order to determine if the biological effect observed is due to the concentrations obtained from the biocomponents.

The bioactive compounds of *Opuntia robusta* fruit (*Or*) have been studied due to its potential beneficial effect on intestinal, cardiovascular, and liver health as well as for having antioxidant and even anticancer properties [14,15,16]. Likewise, this fruit has been used to treat degenerative and metabolic diseases caused by oxidative stress [17].

Previously, the antioxidant and hepatoprotective effects of *Or* extracts on oxidative stress-induced acute liver damage induced by acetaminophen [12,18] and diclofenac intoxication [19] have been reported. Because oxidative stress is a common phenomenon in acute and chronic liver damage caused by different noxious agents, including drugs, chemicals and viruses that can lead to fibrosis, the aim of this study was to determine the nutraceutical properties and the antioxidant, hepatoprotective and antifibrotic effects of *Or* in a rat model of hepatic fibrosis induced with TAA.

## 2. Results and Discussion

### 2.1. Nutraceutical Characterization

#### 2.1.1. Fruit Material

**Figure 1:**
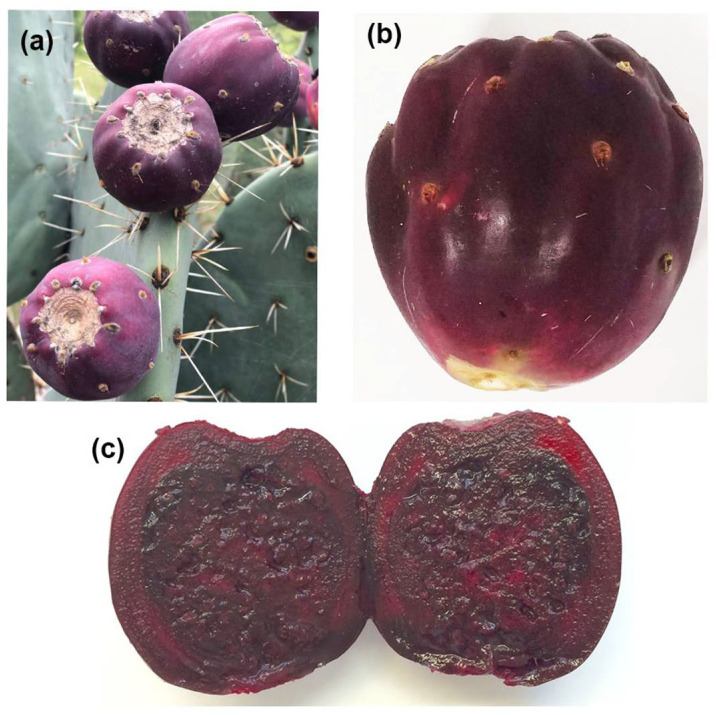
(**a**) Cladodes of *Opuntia robusta* in the wild where they were collected, with some fruits (prickly pears). The cladodes are circular in shape and bluish in color. (**b**,**c**) Ripe fruits reach a size of 7 to 11 cm.

A total of 42.6 kg of prickly pears (Figure 1) were collected, of which 24.5 kg were destined for the extraction of *Or* pulp (*OrP*) and 18.1 kg for the extraction of *Or* extract (*OrE*), obtaining 6.35 and 4.96 L, respectively. The average weight yield of *OrP* obtained was 47.81% (data not shown) and the percentage of lyophilization yield was 13.38% on average.

It has been reported that 28 to 58% of the entire weight of the *Opuntia* fruits is pulp [20], which is consistent with our results.

#### 2.1.2. Determination of Moisture Content

The moisture content of *OrP* was found to be 89.96 ± 0.1% according to the AOAC protocols [21]. The edible portion of the fruit, *OrP*, is composed of 84 to 90% water [20]. A moisture percentage of 84.10 ± 0.14 has been reported previously for *OrP* [22] a figure very close to the one obtained in the present study and determined following AOAC protocols. These species are well known for mitigating food and water shortages in drought-prone areas [23].

#### 2.1.3. Bromatological Analysis of *OrP*

The bromatological analysis of *OrP* is presented in Table 1. We infer from this analysis that the plants from which the fruits were collected are middle-aged because there is an inverse correlation between the crude fiber content, which increases with the age of the plant [24] and the crude protein content that decreases in ageing plants. Moreover, an important nutritional value is inferred from *OrP*, considering its high percentage of nitrogen-free extract (NFE). NFE represents highly digestible carbohydrates, which, like total carbohydrates, also increase with age [24]. All these determinations were made both on dry matter basis (DMB) and on wet matter basis (WMB).

#### 2.1.4. Betacyanins

Betalains are secondary metabolites classified into two main groups: betacyanins and betaxanthins depending on the groups conjugated to the precursor betalamic acid [25]. Betacyanins are considered as antioxidants due to their capability to scavenge free radicals directly by the presence of a cyclic amino group, which acts as a hydrogen donor or indirectly via redox-sensitive transcription factors, such as Nrf2, which activates the cellular antioxidant response via AREs (antioxidant response elements). The content of betacyanins obtained from *OrP* was 436.5 ± 57 mg of betacyanin equivalents/L (Table 2) similar to the values previously reported by González-Ponce et al. (2020) [18], 464.9 ± 10.87 mg of betacyanin equivalents/L and higher than those found by González-Ponce et al. (2016) [12] 333.27 ± 11.46 mg of betacyanin equivalents/L. Taking into account that the weight of 1 L of *OrP* is 1032.6 g, its percentage of humidity is 89.96% and its percentage of solids is 10.04%, the dry basis figure of betalains obtained was 4.18 mg of equivalents of betacyanins per gram of dry *OrP*, a figure significantly higher than that reported by Castellanos-Santiago and Yahia (2008) [7] (2.06 ± 0.06 mg/g dry pulp) for *Opuntia robusta*, which, in turn, was compared with other species: *ficus-indica* (0.39 ± 0.03 mg/g dry pulp), *megacantha* (0.065 ± 0.01 mg/g dry pulp) and *albi-carpa* (0.05 ± 0.02 mg/g dry pulp) species. This indicates that *Or* has a higher antioxidant capacity than some other fruits studied and even higher than other *Opuntia* species because this capacity is directly proportional to their content of betacyanins.

Betanin is an important betacyanin and has a chemical structure that allows the neutralization of free radicals that are mainly responsible for lipid peroxidation. The redox potential of betanin makes this molecule an efficient reducer of peroxyl radicals derived from unsaturated lipids of biological membranes [26]. Betanin also acts as a scavenger of nitrogen dioxide, the radical initiator of the oxidative process of low-density lipoproteins (LDL). The antioxidant capacity of betanin is linked to its phenol group [27] and induces the antioxidant Nrf2 pathway [28]. The molecular structure of betacyanins has an electron-donating site, rich in electrons and an electron-accepting site, which is deficient in electrons, bound by a conjugated π system. This generates an intramolecular electron transfer process and facilitates the flux of electrons within the molecule [29]. The antioxidant potency of betacyanins can be attributed to this mechanism since it can donate or remove electrons from radical species. Betacyanins can reduce the oxidative damage present in hepatic fibrosis induced by thioacetamide by neutralizing superoxide radicals that are generated by its biotransformation.

#### 2.1.5. Total Soluble Phenols

*OrP* has 1118.0 mg GAE/100 g of total soluble phenols (Table 2). Fernández-López et al. (2010) [30] reported 218.8 mg/100 g in *ficus-indica* and compared it with *Opuntia stricta* (204.4 mg/100 g) and *ondulata* (164.6 mg/100 g). *Or* has a significant number of phenolic compounds, even higher than those reported for these species using the same Folin–Ciocalteu method. Phenolic compounds, also known as hydrophilic antioxidants, are the most abundant secondary metabolites in fruits [31]. It has been reported that the pulp of the *Opuntia* spp. fruits contains phenolic compounds and other antioxidants that have a positive effect on the cellular redox balance, mainly due to the reduction of LDL levels [32]. *Or* contains a higher quantity of total soluble phenols than the other *Opuntia* species that have been studied. Moreover, the inhibition of peroxidation of polyunsaturated fatty acids correlates with the antioxidant capacity of polyphenols [33]; consequently, it is inferred that *Or* prevents the oxidation of membrane lipids and, consequently, cell necrosis.

#### 2.1.6. Total Soluble Flavonoids

*OrP* contained 793 mg of catechin equivalents (CAE) per 100 g of fruit on dry basis (Table 2). Part of the beneficial activity of these species is attributed to the synergistic effect of flavonoids with betalains [32]. This suggests that the important antioxidant activity of *OrP* is not only due to its considerable amount of betacyanins, but also from their interaction with phenolic compounds, including flavonoids, which has effects on lipid metabolism [34], increasing plasma levels of eicosapentaenoic acid (EPA) and docosahexaenoic acid (DHA) [35]. Furthermore, the consumption of flavonoids stimulates the conversion of alpha-linolenic acid into long-chain polyunsaturated fatty acids n-3 EPA and DHA [36]. Flavonoids intake has also been associated with an increase in the concentration of polyunsaturated fatty acids by modifying the enzymatic activity or gene expression of the fatty acid desaturase enzymes Δ5 and Δ6 [37]; thus, reducing oxidative damage to membrane lipids. Flavonoids also attenuate oxidative damage by increasing the activity of endogenous antioxidant mechanisms, such as the transcription factor Nrf2 [38].

An excessive generation of ROS, causes oxidation of polyunsaturated fatty acids that constitute biological membranes, eventually causing cell damage and cell death. Flavonoids and betanin inhibit the formation of alkyl radicals, the molecular entity associated with the oxidation of lipids [39]. In addition, betanin has been reported to scavenge superoxide anions [40] and this scavenging effect is augmented in the presence of flavonoids [32].

*Or* has proven to be the species with the highest concentration of betalains, total phenolic compounds and flavonoids [11,12], which confirms its important biological activities, mainly its antioxidant capacity. We propose that their consumption reduces the severity of liver fibrosis.

#### 2.1.7. Antioxidant Capacity

##### DPPH (2,2-Diphenyl-1-picrylhydrazyl)

To evaluate the antioxidant effect and elucidate the possible mechanisms of cytoprotection of *Or*, we performed the DPPH free radical scavenging assay, obtaining 2.27 mmol of Trolox^®^ equivalents (TE)/L for *OrP* (Table 3). González-Ponce et al. (2016) [12] has reported higher figures (5.77 ± 0.33 mmol eq. Trolox/L) for *robusta*; however, they also report a lower figure for the *streptacantha* species (1.31 ± 0.94 mmol eq. Trolox/L).

It has been established that the *Opuntia* fruit species with the highest values in this assay are those with red-purple pigments [41]. This strengthens the evidence that antioxidant activity is closely related to the presence of betalains and their interaction with other biocomponents, such as flavonoids, phenolics and vitamins. In support, DPPH correlates strongly with the amount of flavonoids and phenolic compounds present in the fruit [42].

Using the DPPH method, it has been evaluated that the antioxidant capacity of titanium dioxide nanofibers increases considerably when betanin is added [43]. Therefore, we conclude that *OrP* has a potent radical scavenging activity due to the presence of multiple bioactive compounds, including betacyanins (Table 2).

##### ABTS^•+^ (2,2′-Azino-bis(3-ethylbenzothiazoline-6-sulfonic Acid) Diammonium Salt)

The Trolox Equivalents Antioxidant Capacity (TEAC) of *OrP* evaluated by the ABTS^•+^ method is 62.2 ± 5.0 μmol TE/g on dry matter basis (Table 3). The extracts of the fruit of *Opuntia streptacantha* (61.69 ± 3 mg eq. ascorbic acid/100 mL) have been compared with those of *Opuntia robusta* (92.62 ± 5 mg eq. ascorbic acid/100 mL) [12], confirming that *O. robusta* has a greater capacity to eliminate ABTS^•+^ radical and demonstrating its high capacity to neutralize radicals via two different mechanisms: single electron transfer (SET) or hydrogen atom transfer (HAT) since the ABTS^•+^ radical can be neutralized by any of these routes [44]. In the absence of phenolic compounds, the ABTS^•+^ radical is stable; however, it reacts with hydrogen donors, such as phenolics, and generates a colorless metabolite of ABTS^•+^ [45], indicating that the major mechanism detected in this assay is HAT (Table 2) due to the presence of phenolic compounds in *Or*.

##### FRAP (Ferric Reducing Antioxidant Power)

The TEAC of *OrP* determined by this method, 80.2 ± 11.7 μmol TE/g on dry matter basis (Table 3), indicates the presence of phenolic compounds (Table 2) that can diminish the conversion of ferrous ion Fe^2+^ to the ferric ion Fe^3+^ inhibiting the Fenton reaction that yields the strongly oxidizing hydroxyl radical. *Or* is known to contain almost three times the TEAC (73.24 ± 3 mg eq. ascorbic acid/100 mL) than *O. streptacantha* (28.82 ± 2 mg eq. ascorbic acid/100 mL) [12]. The FRAP assay is specific for the electron donor mechanism (SET) [46]. That is why it is convenient to combine ABTS^•+^ and FRAP techniques to obtain a complete picture of antioxidant capacity of *Or* [44].

##### AAPH (2,2-Azobis(2-amidinopropane) Dihydrochloride)

We determined that 50% of the erythrocyte membrane lipid peroxidation caused by AAPH was reversed by *OrP*, a percentage comparable to that reported for carotenoids (48%) [47]. The capacity of betanin and its aglycone betanidin to inhibit lipoperoxidation and the degradation of the heme group in vitro, even at very low concentrations, has been reported before [48]. Furthermore, betalains are known to inhibit peroxynitrite-mediated tyrosine nitration and DNA chain division [49]. Thus, the antioxidant capacity of these pigments is not limited to a single mechanism of action.

In the third phase of the assay, it was necessary to dilute the *OrP* extract since the concentrated extract exceeded the pre-established values of the vitamin E analogue Trolox (Table 3), suggesting that the synergy of multiple components of *OrP* enhances the antioxidant capacity of each of them, including vitamin E.

##### Capacity to Scavenge H_2_O_2_

The capacity of *OrP* to scavenge H_2_O_2_ was 15 ± 0.8% at 100 µg/mL (Table 3) similar to that of *Crataegus monogyna* water and ethanol extracts (15.44–30.13%) [50]. The ability to neutralize reactive species, such as hydrogen peroxide, can be attributed to the presence of primary antioxidants, such as phenols and flavonoids [51], and these antioxidants are present at significant concentrations in *OrP* (Table 2). Furthermore, betanin pretreatment of HT29 cells significantly prevents DNA damage caused by H_2_O_2_ [40] and it has been reported that betanin protects DNA against ROS and RNS [40,49].

##### Vitamins E and C

**Figure 2:**
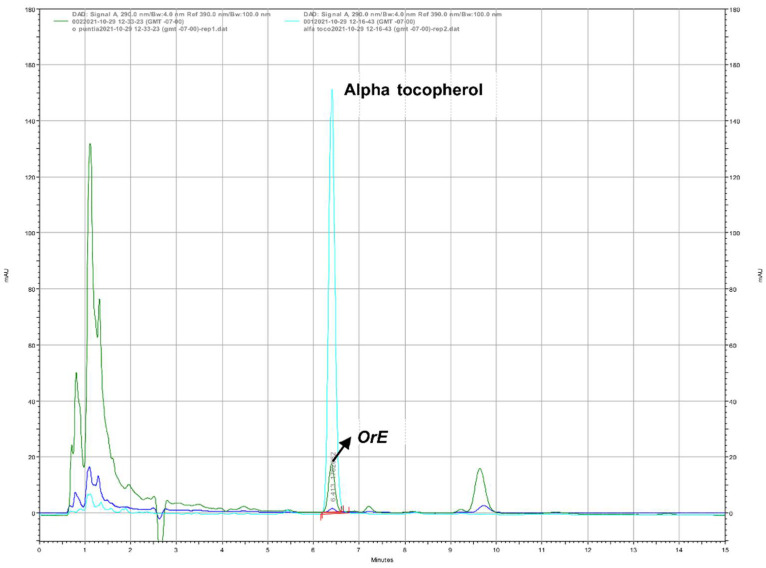
HPLC-UV chromatogram of alpha tocopherol standard and vitamin E in *Opuntia robusta* extract (*OrE*).

**Figure 3:**
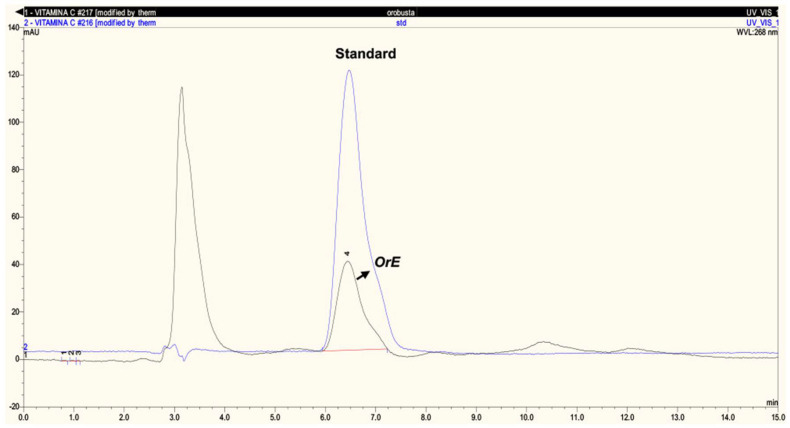
HPLC-UV chromatogram of vitamin C in *OrE*.

Vitamins E and C are potent antioxidants present in *OrE* at 429.9 μg/100 g and 141.14 mg/100 g, respectively (Figure 2 and Figure 3). The content of vitamin C was very similar to that reported for guava, a fruit known for its significant content of vitamin C (136.99 mg/100 g) [52].

The ability of these vitamins to eliminate ROS has been reported and there is a direct correlation between their concentration and the preservation of reduced glutathione (GSH) levels [53]. Furthermore, the gene expression of glutathione reductase and glutathione peroxidase are increased by their intake [53,54]. The concomitant administration of *OrP* or *OrE* with TAA increases GSH levels (F indicating a beneficial effect of the components of the fruit, including vitamins E and C. The beneficial effects on the levels of non-enzymatic antioxidants provided by the intake of vitamins E and C have been reported previously in type II diabetes mellitus (DMT2) when the vitamins were used as dietary supplement [54].

Considering that the oxidation of membrane lipids generated by the abstraction of hydrogen atoms is one of the most important mechanisms of damage caused by TAA and that vitamin E represents one of the most important fat-soluble antioxidants that provides protection to lipoproteins and phospholipids of biological membranes and stored lipids [55], this vitamin could mitigate the hepatic damage caused by TAA via hydrogen donation or HAT mechanism. Using the DPPH, ABTS^•+^ and AAPH techniques, we have demonstrated in this study that *OrP* has the capacity to reduce lipoperoxidation via these mechanisms.

Vitamin E reacts with peroxyl radicals of fatty acids, primary products of lipid oxidation, preventing their subsequent reaction with macromolecules. This vitamin has a beneficial effect on chronic liver damage, which is characterized by sustained inflammation, since it can reduce this biological response [56].

On the other hand, vitamin C modulates NF-kB nuclear translocation and reduces the signaling cascade that leads to HSC activation, collagen deposition and, ultimately, liver fibrosis [57].

Because vitamins E and C are exogenous micronutrients not associated with toxicity, as well as free radical scavengers and lipid peroxidation inhibitors, they should be included in the diet. This study demonstrates their presence in *Or* fruits.

Almost all the assays used in this study evaluate the HAT mechanism (except ABTS^•+^ that evaluates both HAT and SET and FRAP that only detects SET). However, there is evidence that electron donation by the betanin molecule is also a mechanism of action [58], which suggests that betanin is a compound that scavenges free radicals and reactive molecules via various mechanisms. One of these mechanisms is radical quenching: it removes superoxide and singlet oxygen radicals via a physical scavenging mechanism [59]. It should be noted that both synergistic and antagonistic effects occur among molecules with different degrees of antioxidant capacity, such as flavonoids, phenolics and vitamins E and C [60].

### 2.2. Animal Studies with Thioacetamide

#### 2.2.1. Animals and Treatments

Fibrosis is the result of chronic damage to the liver parenchyma and is characterized by the elevation of aminotransferases, increased oxidative stress and alterations in hepatic architecture. Using markers to assess these changes in the liver, we determined the effects of *Or* on fibrogenesis induced by TAA.

#### 2.2.2. Liver Damage Markers

**Figure 4:**
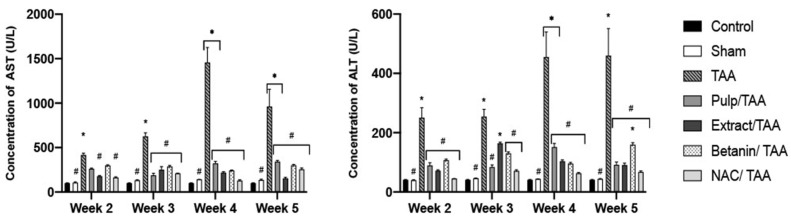
Aspartate aminotransferase (AST) and Alanine aminotransferase (ALT) concentrations for different treatments at different evaluation times. Two-way ANOVA with Tukey’s post hoc test. * *p* < 0.05 compared to control group, ^#^ *p* < 0.05 compared to thioacteamide group.

High serum levels of ALT and AST are indicators of liver damage [56]. Chronic administration of TAA causes elevation of serum aminotransferases [61]. The administration of *OrP* significantly reduced ALT and AST levels in TAA-treated animals, indicating reduced liver damage. In the second week of treatment, *OrP* reduced ALT levels more than betanin (Figure 4). Showing that the other biocomponents of *OrP* act synergistically with the betacyanins in the fruit (Table 2), providing increased cytoprotection to hepatocytes.

During the third week, serum AST levels in rats from the *OrP*/TAA group did not differ from the control group, but did differ from the TAA group. The serum levels of ALT in the *OrP*/TAA group were lower than in the *OrE*/TAA and betanin/TAA groups and similar to the ALT levels in the NAC/TAA group (Figure 4). *OrP* decreased hepatocyte damage very effectively in the early stages of the pathogenesis of liver fibrosis. After four weeks of treatment, the experimental groups *OrE*/TAA and NAC/TAA showed significant differences compared to the TAA group (Figure 4). In contrast, the protection provided by *OrP* is very limited at this stage.

Finally, in the fifth week, the serum AST levels of all the treatment groups demonstrated a significant difference compared to the TAA group (Figure 4), indicating significant protection against the lesions caused by TAA. *OrP* and *OrE* also significantly reduced ALT levels compared to the TAA group (Figure 4), showing a marked protection to hepatocytes, since they are the cells that mainly produce this aminotransferase.

#### 2.2.3. Oxidative Stress Markers

##### GSH (Reduced Glutathione)

**Figure 5:**
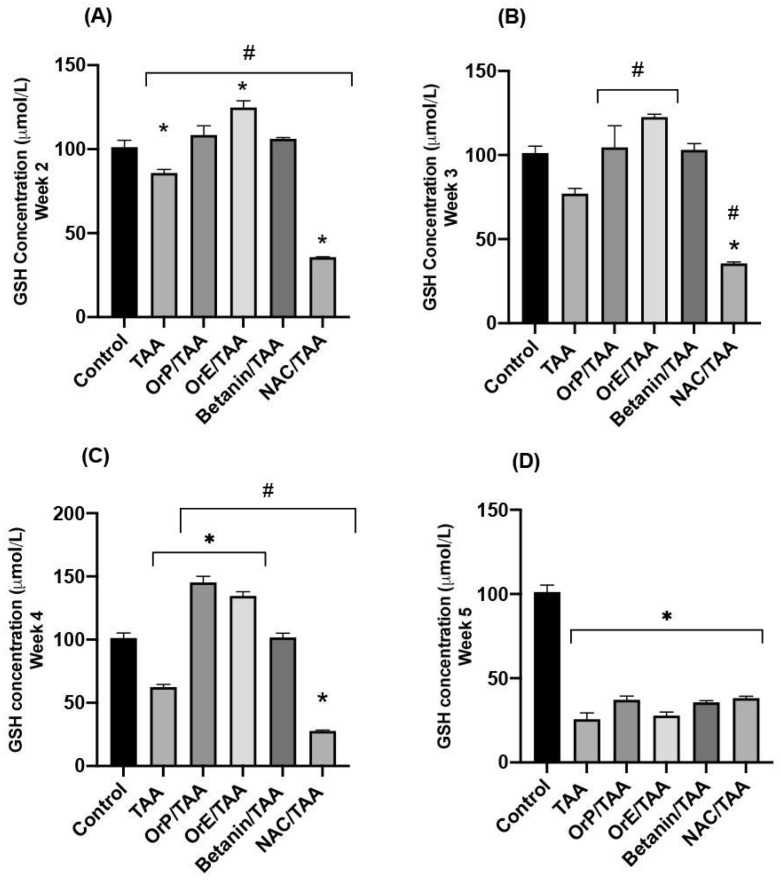
Concentrations of reduced glutathione (GSH) in liver tissue for the different treatments at different evaluation times. (**A**–**C**) *OrP* and *OrE* help to restore GSH levels. (**D**) A significant depletion of GSH is generated, the oxidative damage is severe in chronic treatment and hepatoprotectors have no effect at this point. One-way ANOVA with Tukey’s post hoc test. * *p* < 0.05 compared to control group, # *p* < 0.05 compared to TAA group.

**Figure 6:**
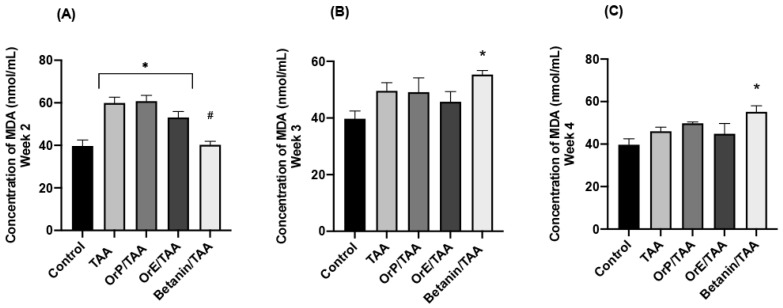
Concentrations of malondialdehyde (MDA) for the different treatments at different evaluation times. (**A**–**C**) *OrE* lowers malondialdehyde levels more effectively than *OrP.* One-way ANOVA with Tukey’s post hoc test. * *p* < 0.05 compared to control group, # *p* < 0.05 compared to TAA group.

**Figure 7:**
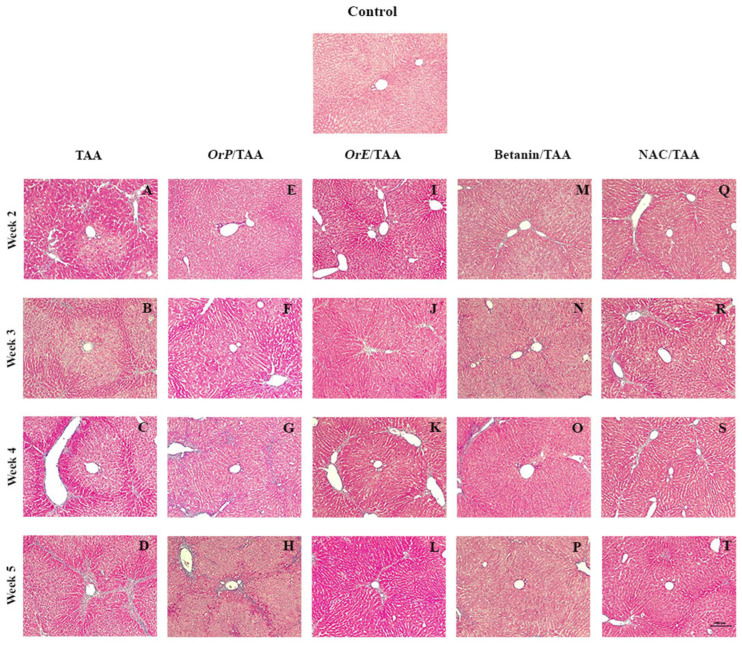
Damage projections from zone 3 to portal spaces. (**A**–**D**) Treatment with TAA showed extensive areas of vacuolation, necrosis, and fibrosis. Concomitant treatments decrease the severity of the damage. (**E**–**H**) *OrP* delays the morphological changes of the fibrotic process generated by TAA. (**I**–**T**) *OrE*, Betanin and NAC decrease the fibrotic process in all weeks of treatment. Masson’s trichrome staining. Magnification (100×).

**Figure 8:**
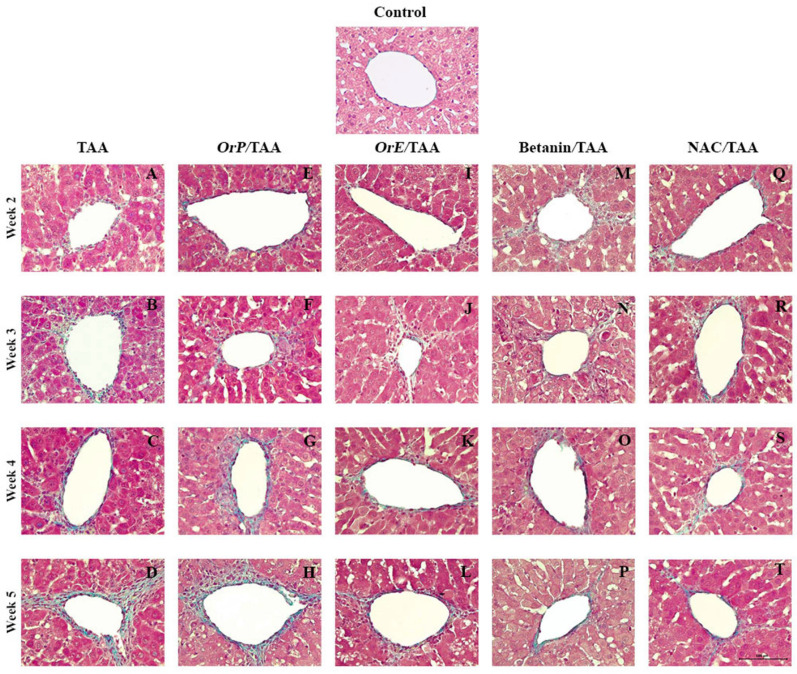
Effect the hepatoprotectors on histopathology in TAA-induced liver damage through time. (**A**–**D**) Necrosis and fibrosis septa are shown in zone 3 of the hepatic acinus with TAA administration. (**E**–**T**) Treatments with *OrP*, *OrE*, Betanin, and NAC decrease the number of damaged cells and the thickness of collagen septa. Masson’s trichrome staining. Magnification: 400×.

Administration of TAA induces oxidative stress in liver tissue via the generation of hydroperoxides during membrane lipid peroxidation, which is reflected in the decrease of hepatic GSH concentrations (Figure 5) as well as the increase in the MDA values (Figure 6) [62] and the activity of glutathione peroxidase [63]. The toxic metabolite of TAA (TASO_2_) acts as an electrophilic compound that increases cellular oxidative stress. TASO_2_ is directly neutralized by glutathione, which is why a significant decrease in reduced glutathione levels is observed at all time points of treatment with TAA (Figure 5A–D). However, when TAA is administered concomitantly with hepatoprotective agents, a higher concentration of GSH is shown even compared to the control group. As mentioned before, betanin is a potent inducer of GSH synthesis via the erythroid nuclear factor 2 (Nrf2) pathway, which demonstrates the important antioxidant capacity of this pigment, since Nrf2 is an important regulator of the antioxidant response. As shown in Figure 5A–C, treatment with betanin restores GSH levels to the levels observed in the control group, while treatment with *Or* seems to even increase tissue concentration of GSH compared to the control group. This increase occurs at all time points of treatment but is greater at week 4. *OrE*, during the second and third weeks of treatment, presented greater cytoprotective effects (Figure 5), in accordance with biochemical and histopathological data (Figure 4, Figure 7 and Figure 8I–L), but at week 4, it is the treatment with *OrP* that promotes synthesis of GSH through redox cycles and/or facilitates the binding of GSH to cellular proteins to delay their oxidation, using *OrP* antioxidant machinery as a priority rather than depleting glutathione stores.

The dose of NAC used in this experimental model was effective in reducing liver damage as reflected in biochemical (Figure 4) and histopathological markers (Figure 7 and Figure 8). However, hepatic GSH levels showed a significant depletion in comparison to the control and TAA groups. There are reports in which this drug has been used for the treatment of early liver injury at lower doses [64] indicating that the dose of the drug is important to achieve the desired antioxidant effect.

In acute acetaminophen intoxication, there is evidence that *Opuntia streptacantha* partially prevents GSH depletion, while *Opuntia robusta* does it completely [12]. In the same study, it was reported that the treatment with *Opuntia* restored GSH concentration very close to the control levels. In the present study, GSH levels in the groups treated with *OrE* and *OrP* were higher than those shown by the control group (Figure 5). It has also been reported that the presence of phenolic compounds from the fruit of *Opuntia robusta* acts in synergy with betacyanins (Table 2), potentiating the antioxidant effect of the compounds compared to when they are isolated [32].

##### MDA (Malondialdehyde)

The excessive formation of superoxide radicals caused by TASO_2_ leads to the oxidation of unsaturated membrane lipids and the subsequent formation of MDA, which forms adducts with DNA, eventually triggering cell death. Likewise, lipid peroxidation of the cell membrane of hepatocytes is one of the main causes of liver injury [65]. Accordingly, in Figure 6A–C, we show that in our experimental model there was an increase in MDA throughout the treatment with TAA from week 2 onwards. High doses of TAA are known to induce oxidative stress and lipid oxidation as well as a decrease in antioxidant status [66].

*OrE* is more protective than *OrP* since it decreased the MDA levels on weeks 2, 3, and 4, while *OrP* did not provide any protection (Figure 6), suggesting the possibility of adhesion of the active components of the fruit to the insoluble fiber, making their availability and absorption more difficult. *OrE*, at weeks 3 and 4 (Figure 6B,C) of treatment, proved to be even more effective than betanin. This suggests that betanin alone only shows protection in the early stages of damage but becomes more protective in the presence of other biocomponents of *OrE* (Figure 6A). In addition, there is evidence to support that toxic products play an important role in betanin oxidation [67]. *OrE* is probably more protective because of the presence of both betalains and flavonoids. *OrE* is also protective in a model of acetaminophen intoxication in the liver and this protection correlates with reduced MDA levels [18].

#### 2.2.4. Histopathological Study

Compared to healthy controls, the livers of rats treated with TAA showed extensive areas of vacuolation, necrosis, and fibrosis, especially in zone 3, inflammatory infiltrate and loss of the normal architecture of the liver parenchyma (Figure 7A–D). It was observed that the administration of *Or* significantly reduced the histopathological changes induced by TAA.

**Figure 9:**
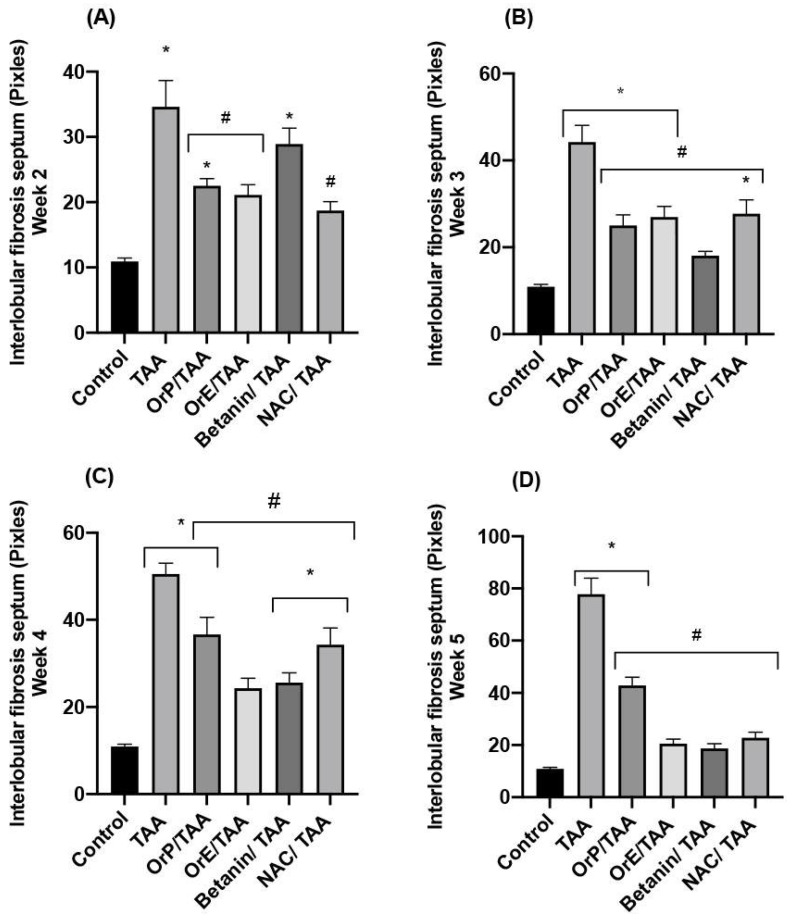
Measurements of interlobular fibrotic septa for the different treatments at different time points. (**A**–**D**) In all the evaluated weeks of treatment, *OrP* and *OrE* significantly decrease the thickness of the fibrosis septa. One-way ANOVA with Tukey’s post hoc test. * *p* < 0.05 compared to control group, # *p* < 0.05 compared to TAA group.

Damage was observed from zone 3 to the portal spaces (Figure 7A–D); this is due to the differential expression of cytochrome P450 enzymes that are responsible for the biotransformation of TAA into TASO_2_ [68]. There is evidence that chronic intoxication with TAA drastically reduces the expression of the hepatic proteins CYP1A2, CYP2C6, CYP2E1, and CYP3A2 by 18, 71, 30, and 21%, respectively, compared to the control group [61]. This pattern of damage was persistent; although, the administration of *OrP* for two weeks (Figure 7E, Figure 8E and Figure 9) attenuated the damage (Figure 7A, Figure 8A and Figure 9).

From the third week of administration of TAA (Figure 7B and Figure 8B), a beneficial effect is observed for *OrP* (Figure 7F and Figure 8F) and *OrE* (Figure 7J and Figure 8J)*,* comparable or even more than observed with NAC (Figure 7R and Figure 8R), a hepatoprotective drug used clinically to restore GSH levels on fibrotic diseases [69]. *OrP* and *OrE* decreases cell damage at the early stages and reduced fibrosis at all stages (Figure 9). At week 4 of *OrP*/TAA treatment (Figure 7G and Figure 8G), the fibrotic process was well developed; although, the surface area of damaged liver parenchyma was less compared to the TAA group (Figure 7C and Figure 8C). Both *OrP* and *OrE* decreased the thickness of the fibrotic interlobular septa, with *OrE* being more effective (Figure 9).

The fibrotic septa caused a total loss of the normal architecture in the liver lobules at week 5 of administration of TAA (Figure 7D). At this time, histologically, *OrP* decreased necrosis and fibrosis (Figure 9) induced by TAA (Figure 7H). Treatment with *OrE* considerably decreased the thickness of the fibrosis septa (Figure 7L, Figure 8L and Figure 9). *Or,* although to a lesser extent, also exerts a cytoprotective effect at advanced stages of liver fibrosis, delaying the formation of fibrotic septa.

Treatment with *OrP* and *OrE* mitigated histopathological changes in the liver of rats receiving TAA and this can be attributed to their antioxidant properties, reduction of MDA levels and improvement of reduced glutathione levels.

## 3. Materials and Methods

### 3.1. Chemicals and Reagents

Thioacetamide, betanin, N-acetylcysteine, GSH/GSSG kit, sodium chloride, procaine, heparin, paraformaldehyde, sodium hydroxide, gallic acid, Folin–Ciocalteu reagent, catechin, ascorbic acid, dithiothreitol (DTT), DPPH (2,2-diphenyl-1-picryl-hydrazyl-hydrate), Trolox^TM^ ((±)-6-hydroxy-2,5,7,8-tetramethylchromane-2-carboxylic acid), sodium acetate, TPTZ (2,4,6-tris(2-pyridyl)-s-triazine), ABTS^•+^ (2,2′-azino-bis(3-ethylbenzothiazoline-6-sulfonic acid) diammonium salt), potassium persulfate, AAPH (2,2′-azobis (2-methylpropionamidine) dihydrochloride), sodium nitroprusside, sulphanilic acid, N-1-naphthyl ethylenediamine were all obtained from Sigma Aldrich (St. Louis, MO, USA). Acetonitrile (nanograde) was from Mallinckrodt. Formaldehyde, absolute methanol and glacial acetic acid were from J.T. Baker (Madrid, Spain). Hydrochloric acid and ferric chloride were from Fermont (Monterrey, México) and sodium pentobarbital was from Pisabental, Pisa Agropecuaria, México.

### 3.2. Nutraceutical Characterization of Opuntia Robusta Fruit

#### 3.2.1. Fruit Material

The *Or* fruits were collected in the summer of 2019–2020 at the community Soledad de Abajo, Aguascalientes, México (21°47′04.7″ N 102°06′40.1″ W). The selection was based on the ripeness and integrity of the fruit, considering 7 to 11 cm purple-colored fruits as mature ones.

After harvest, the fruits were washed, disinfected and the glochids were removed. After hand-peeling, the seeds were separated; thus, obtaining *OrP*; the prickly pears destined to obtain *OrE* were centrifuged at 5000× rpm for 20 min and filtered. *OrP* and *OrE* were lyophilized and stored at −80 °C until analyses and treatments.

#### 3.2.2. Determination of Moisture Content of *OrP*

The moisture content was analyzed according to standard protocols [21].

#### 3.2.3. Bromatological Analysis of *OrP*

The bromatological analysis of *OrP* was performed at the Zootechnical Station of Universidad Autónoma de Aguascalientes following the recommended protocols of the AOAC [70].

#### 3.2.4. Betacyanins

The evaluation of the betacyanin content was performed according to González Ponce et al. (2020) [18] with modifications. Dilutions of 1:30 and 1:40 of *OrP* were monitored at 538 nm. The following equation was applied for each value:

(1) $$\mathrm{BT}\;\left[\frac{\mathrm{mg}}{L}\right]=\frac{A*\;\mathrm{FD}*\;\mathrm{MW}*\;1000}{\varepsilon \;*\;l}$$

where, “A” is the sample absorbance, “FD” the dilution factor, “MW” the molecular weight (550 g/mol), “ε” the molar extinction coefficient [60,000 L/mol·cm in water], and “l” the cell read length.

#### 3.2.5. Total Soluble Phenols

Based on the methodology described by Takoudjou Miafo et al. (2022) [71], 25 mL of the sample methanolic extract was mixed with 125 µL of 2 N Folin–Ciocalteu phenol reagent and, after 6 min, 7% sodium carbonate was added. The reaction volume was brought to 3 mL with distilled water and incubated for 90 min at room temperature. Samples were centrifuged (Micro 2000 Clay Adams™ Becton Dickinson microcentrifuge) at 12,000× rpm for 3 min and read at 757 nm in a Biomate 3 Thermo Scientific spectrophotometer. The content of total soluble phenols of the sample was calculated as gallic acid equivalents using the equation shown below. The results were extrapolated from a gallic acid calibration curve.

(2) $$\mathrm{GAE}=\left[\frac{A-b}{m}\right]\times \left(\frac{\mathrm{Vt}}{\mathrm{Va}}\right)\times \left[\frac{1}{\mathrm{Mh}\times 10}\right]\times \left[\frac{1}{100-H}\right]$$

where, “GAE” is the milligrams of gallic acid equivalents/gram of dry sample, “A” the absorbance of the sample tube, “b” the intercept (absorbance), “m” the slope (absorbance/microgram gallic acid), “Vt” the total volume of the extract (microliters), “Va” the volume of aliquot used in the test tube (microliters), “Mh” the weight of the extracted wet sample (grams) and “H” the moisture content of the wet sample (percent).

#### 3.2.6. Total Soluble Flavonoids

According to Ilyas et al. (2022) [72], a volume of 500 μL of methanolic *OrP* extract was mixed with 5% (*w*/*v*) sodium nitrite, 10% (*w*/*v*) aluminum chloride and 1 M sodium hydroxide. A final reaction volume of 2.5 mL with distilled water was incubated for 30 min, protected from light. The samples were read at 510 nm. A calibration curve was made with catechin at different concentrations. The content of total soluble flavonoids of the sample was calculated as catechin equivalents using Equation (2).

#### 3.2.7. Antioxidant Capacity

##### Methanolic Extract

The antioxidant capacity of *OrP* was evaluated with a methanolic extract obtained according to the method described by Lasano et al. (2019) [73], except in the AAPH method, in which we use PBS.

##### DPPH (2,2-Diphenyl-1-picrylhydrazyl)

According to the method used by Ruslan et al. (2018) [74], 75 μL of the methanolic *OrP* extract was mixed with acidic (pH 2) absolute methanol-70% aqueous acetone 1:1 (*v*/*v*) (MAA) to 100 μL. Then, 600 μL of DPPH 0.13 mM was added and, after incubating the samples for 20 min, protected from light, they were read at 515 nm, having calibrated to zero absorbance with an absolute methanol blank. The result of the test samples was calculated by simple linear regression analysis and expressed as antioxidant activity equivalent to Trolox^TM^ per unit weight of the wet or dry sample, using Equation (2) and extrapolating form a Trolox^TM^ calibration curve.

##### ABTS^•+^ (2,2′-Azino-bis (3-ethylbenzothiazoline-6-sulfonic Acid) Diammonium Salt)

In accordance with Xu et al. (2022) [75], 50 µL of *OrP* was used in triplicate and MAA was added to a final volume of 150 µL. Subsequently, 2850 μL of ABTS^•+^ solution (ABTS + potassium persulfate) was added. After incubation for 2 h in the dark, the absorbance of the samples was measured at 734 nm having zero absorbance calibrated with an absolute methanol blank. The result of the test sample was calculated by simple linear regression analysis and expressed as antioxidant activity equivalent to Trolox^TM^ per unit weight of the wet or dry sample, using Equation (2) and extrapolating from the Trolox^TM^ calibration curve.

##### FRAP (Ferric Reducing Antioxidant Power)

According to Khudyakov et al. (2022) [76], the calibration curve was made using 0.666 mM Trolox^TM^. Fifty microliters of the biological sample were used, and MAA was added to a final volume of 150 μL, followed by the addition of 2850 μL of FRAP solution. The tubes were protected from light for 30 min at room temperature and the samples were read at 593 nm. The spectrophotometer was calibrated to zero absorbance with a blank tube without Trolox™. The result of the test sample was calculated with Equation (2) by simple linear regression analysis and expressed as antioxidant activity equivalent to Trolox™ per unit weight of the wet or dry sample.

##### AAPH (2,2-Azobis(2-amidinopropane) Dihydrochloride)

Four milliliters of human blood were centrifuged at 3500× rpm for 5 min to remove the plasma and the buffy coat. Five washes were carried out with PBS pH 7.4, discarding the supernatant each time. A 5% (*v*/*v*) suspension of erythrocytes was prepared in PBS pH 7.4.

After confirming the lack of lytic capacity of the *OrP* extract on erythrocytes, using the methodology described by Vinjamuri et al. (2015) [77], the analysis of its cytoprotective capacity based on the method described by Chisté et al. (2014) [47] was performed using AAPH as a membrane oxidizing agent. The determinations were made on a 1:10 *OrP* dilution (or PBS pH 7.4), a 5% (*v*/*v*) erythrocyte suspension and a 150 mM AAPH solution (except for the blanks). The tubes were incubated at 37 ± 0.5 °C with orbital shaking at 200 rpm for 5 h in darkness, then centrifuged at 685× *g* for 5 min at 25 °C and their supernatants were read at 535 nm. The following equation was applied to determine the hemolysis inhibition percentage of *OrP*:

(3) $$\%\;\mathrm{of}\;\mathrm{inhibition}\;\mathrm{hemolysis}=100-\left[\frac{\left(\mathrm{Amp}-\mathrm{Acn}\right)}{\mathrm{Acp}-\mathrm{Acn}}\right]$$

where, “Amp” is the absorbance of the sample, “Acn” is the absorbance of the negative control and “Acp” is the absorbance of the positive control.

A standard curve with known quantities of Trolox^TM^ was made and the following polynomial equation was solved using the Wolfram Alpha^®^ software:

(4) $$y=ax^{3}\cdot bx^{2}+cx+d$$

Using Equation (5), the sample values expressed in Trolox equivalents were obtained.

(5) $$\mathrm{Eq}.\;\mathrm{Trolox}=\left(\frac{\mathrm{Abs}-b}{m}\right)\left(\frac{\mathrm{Vt}}{\mathrm{Va}}\right)\left(\frac{1}{Mh\times 10}\right)\;\left(\frac{1}{100-H}\right)$$

where, “Eq. Trolox” is the micromoles of Trolox equivalents/gram of dry sample, “A” is the average absorbance of the replicates of the test sample, “b” is the intercept (absorbance), “m” is the slope (absorbance/nanomole of Trolox), “Vt” the total volume of the extract (microliters), “Va” the volume of aliquot used in the test tube (microliters), “Mh” the weight of the extracted wet sample (grams) and “H” the moisture content of the wet sample (percent).

##### Capacity to Scavenge Hydrogen Peroxide (H_2_O_2_)

According to the method adopted by Khan et al. (2022) [78], different concentrations of *OrP* were added to 40 mM hydrogen peroxide solution (0.6 mL). After 10 min, the absorbance of the hydrogen peroxide at 230 nm was read against the blank solution containing the phosphate buffer without the hydrogen peroxide. Subsequently, the hydrogen peroxide removal percentage was calculated from the following equation.

(6) $${\%\;H}_{2}O_{2}\;\mathrm{scavenged}=\left[\left(A_{c}-{\;A}_{s}\right)/A_{c}\right]\times 100$$

where, A_c_ is the absorbance of the control and A_s_ is the absorbance in the presence of the sample of Opuntia robusta extracts or standards.

##### Determination of Vitamins C and E by High Performance Liquid Chromatography-UV (HPLC-UV)

**Vitamin C**

The determination of ascorbic acid (vitamin C) was performed using the technique established by Singh et al. (2016) [79] in a Thermo Scientific ACCELA PDA Detector ACCELA 600 pump. The preparation of the ascorbic acid standard was achieved by stirring it for 5 h at room temperature in ethanol with activated charcoal and air was continuously bubbled through the solution. Both standards and *OrE* samples were diluted to optimal levels with a mobile phase containing tyrosine (50 mg/100 mL). Dithiothreitol (DTT) was subsequently added to a final concentration of 100 mg/100 mL. The weak anion exchange mode was the method by which the optimal separations were observed. Waters µBondapak NH_2_ 10 mm 3.9 × 300 mm columns with acetonitrile mobile phase: 0.05 M KH_2_PO_4_ (75:25 *w*/*w*) at a flow rate of 1.0 mL/min were used and the volume of injection was 10 µL. The KH_2_PO_4_ solution was heated and kept at 40 °C during the acetonitrile addition, to avoid KH_2_PO_4_ precipitates when acetonitrile was mixed with aqueous solutions. Ascorbic acid was detected by UV at 268 nm (detection limit 25 ng).

**Vitamin E**

The determination of vitamin E in *OrE* was carried out according to Limbach et al. (2021) [80] using a diode array detector Agilent 1260 and with the following specifications: analytical column (Microsorb 100-3 C18 100 × 4.6 mm) at an elution flow rate of 1.0 mL/min. Twenty microliters of the sample were injected. Sample cycle: 30 min. The mobile phase was isocratic [methanol: water (98:2, *v*/*v*)] and readings were made at a wavelength of 290 nm. Alpha-tocopherol was used as standard.

### 3.3. Experimental Groups

#### 3.3.1. Animals and Treatments

Male Wistar rats of 120–150 g were divided into 11 experimental groups: Control, Sham, TAA, *OrP*, *OrE*, Betanin, N-acetylcysteine (NAC), *OrP*/TAA, *OrE*/TAA, Betanin/TAA, NAC/TAA. TAA was dissolved in 0.9% physiological saline solution and administered intraperitoneally twice a week; the first week 250 mg/kg and the following ones 200 mg/kg [81]. The test substances were dissolved in distilled water and administered daily. NAC was dissolved in 0.9% physiological saline solution and administered intraperitoneally at 50 mg/kg. *OrP* and *OrE* orally at 800 mg/kg (12,18) and betanin orally at 25 mg/kg [82] using a stainless steel curved oesophageal cannula (18 × 3″ Cadence Science). The treatments were administered for 5 weeks, and samples of blood and liver tissue were taken for analysis at the end of the 2nd, 3rd, 4th and 5th weeks. All animals had free access to food and water during the experiment and their body weights were monitored every week.

At the end of the 2nd, 3rd, 4th and 5th week, eight rats from each group were anesthetized with sodium pentobarbital (40 mg/kg), then a thoracotomy was performed, and blood samples were taken by cardiac puncture, centrifuged at 3500× rpm for 15 min to obtain serum and then stored at −80 °C until the evaluation of biomarkers of liver damage. The livers of 4 rats were perfused with lavage solution and a total hepatectomy was performed. Liver samples were preserved at −80 °C until the evaluation of markers of oxidative stress. The livers of the remaining four individuals in each experimental group were fixed with neutral formalin (*v*/*v* reagent grade formalin/phosphate buffer) after perfusion with lavage solution and total hepatectomy to perform the histopathological study. Liver tissue samples were placed in neutral formalin until processing.

Our research protocols were approved by the ethics committee of Universidad Autónoma de Aguascalientes for the in vivo experiments and were performed following the guidelines of Mexican government NOM-062-ZOO-1999 and the guidelines of the National Institutes of Health for the care and use of laboratory animals (NIH publications no. 8023).

#### 3.3.2. Biomarkers of Liver Damage

The concentrations of the enzymes aspartate aminotransferase (AST) and alanine aminotransferase (ALT) in blood serum were determined via dry chemistry in the VITROS^®^ System Integrated 5600 Ortho Clinical Diagnostics Johnson & Johnson Company equipment, Neptune Township, NJ, USA.

#### 3.3.3. Oxidative Stress Biomarkers

##### GSH (Reduced Glutathione)

GSH levels were determined in liver tissue after homogenization in the TissueLyser II QIAGEN^®^ equipment, Hilden, Germany. The ratio of oxidized glutathione, glutathione disulfide (GSSG), to GSH was determined using a commercial kit (Quantification kit for oxidized and reduced glutathione, Sigma Aldrich. St. Louis, MO, USA). Determinations were performed on a 96-well plate in an iMark^TM^ Microplate Absorbance Reader BIO-RAD. Hercules, CA, USA at 415 nm.

##### MDA (Malondialdehyde)

MDA concentrations were determined in liver tissue after homogenization using the TissueLyser II QIAGEN^®^ equipment. The methodology described by González-Ponce et al. (2020) [18] was used and the samples were read on a HACH DR/4000U spectrophotometer at 530 nm.

#### 3.3.4. Histopathological Study

Tissue fragments were embedded in paraffin. Sections were cut at 5 μm and processed as previously described [83] to assess hepatic morphology. Liver fibrosis was assessed using Masson’s trichrome staining. Three slides per individual were evaluated under a microscope from Primo Star, Zeiss.

#### 3.3.5. Measurements of Interlobular Fibrotic Septa

We performed the measurements of the fibrotic septa in the interlobular space on 3 different fields per slide of 3 individual rats per time for each of the treatments. A photomicroscope Leica DM750, camera ICC50W. Heerbrugg, Switzerland and the LAS EZ version 3.4.0 software Leica Microsystems, Switzerland were used to take the pictures and the Axio Vision Rel. 4.6 software. Oberkochen, Germany to make the measurements. We determined the areas of collagen in the interlobular zone as pixels at a total magnification of 100×. Parts that did not show the blue staining pattern characteristic of collagen of Masson’s trichrome staining were excluded. The results were expressed as the total number of positive pixels for collagen.

#### 3.3.6. Statistical Analysis

For the determination of the moisture percentage, results were expressed as mean ± standard deviation; n = 3. The bromatological analysis was carried out from a pool of 5 fruits and the results correspond to this analyzed sample. Betacyanin results were expressed as mean ± standard deviation; n = 3. For phenolic compounds and flavonoids, different aliquots of the extract were used (250 μL). Results of the antioxidant capacity determination were expressed as mean ± standard deviation; n = 3.

For the determination of biochemical markers of liver damage, the results were analyzed using two-way ANOVA with Tukey’s post hoc test using GraphPad Prism 9.0.1 software. San Diego, CA, USA with a significance of *p* < 0.05. Results represent the mean of each group (n = 4) ± standard error of the mean (S.E.M.).

For oxidative stress markers (GSH n = 4; MDA n = 6) and for the measurement of interlobular fibrotic septa (n = 9) the results were analyzed using one-way ANOVA with Tukey’s post hoc test using GraphPad Prism 9 software. San Diego, CA, USA with a significance of *p* < 0.05. Results represent the mean of each group ± standard error of the mean (S.E.M.).

## 4. Conclusions

Our results showed that *Or* contains biocomponents with nutraceutical and antioxidant capacity that prevent hepatocyte damage and death, attenuates the exacerbated deposition of extracellular matrix components in the space of Disse and modulates the sustained inflammation in the pathogenesis of liver fibrosis. The donation of electrons by the betanin molecule, the presence of vitamins E and C, the restoration of GSH levels, in addition to the donation of hydrogen atoms, are some of the possible mechanisms by which *Or* exerts its beneficial effect. The antioxidant mechanisms involved are mediated by the components contained in the fruit, being more effective in the extract than in the pulp.

*Or* delays GSH depletion, decreased MDA levels and protects cell integrity, which is reflected in the reduction of serum ALT and AST levels and the significant reduction of tissue inflammation, necrosis, vacuolation, and fibrotic septa.

This study demonstrates that the treatment with the extract and pulp of *Opuntia robusta* ameliorates the characteristics of liver fibrosis. Its concomitant consumption with TAA can attenuate the fibrosis process, in a similar way as NAC, a drug used clinically in the treatment of fibrosis. We propose the intake of the fruit to reduce the risk of developing liver fibrosis/cirrhosis. However, detailed molecular studies are pertinent to determine the exact mechanism by which *Opuntia robusta* exerts its beneficial effect.

## Figures and Tables

**Table 1:** *OrP* (*Opuntia robusta* pulp) bromatological analysis.

Component (%)	DMB	WMB
Dry matter	100	37.99
Crude protein	1.18	0.45
Crude fiber	2.84	1.08
Crude fat	0.76	0.29
Ashes	6.75	2.56
Nitrogen free extract	88.47	33.61

The percentages are given on dry matter basis (DMB) and wet matter basis (WMB).

**Table 2:** Quantification of betacyanins, total phenolic compounds and flavonoids in *OrP* (*Opuntia robusta* pulp); GAE/g = mg gallic acid equivalents/gram, dry matter basis (dmb); CAE/g = mg catechin equivalents/gram, db.

Betacyanins	Total Phenols	Flavonoids
436.5 ± 57 mg of betacyanin equivalents/L	1118.0 mg GAE/100 g, dmb	793 mg CAE/100 g, dmb

**Table 3:** TEAC (Trolox Equivalents Antioxidant Capacity) of *OrP* by different methods and its capacity to scavenge H_2_O_2_; μmol TE/g, db = μmol of Trolox^®^ equivalents per gram of dry sample. Values are the means of three repetitions.

DPPH	ABTS^•+^	FRAP	AAPH	Capacity to Scavenge H_2_O_2_
2.27 mmol TE/L	62.2 ± 5.0 μmol TE/g, db	80.2± 11.7 μmol TE/g, db	247.9 ± 15.6 μmol TE/g, db	15 ± 0.8% at. 100 µg/mL

DPPH: 2,2-Diphenyl-1-picrylhydrazyl; ABTS^•+^: 2,2′-azino-bis (3-ethylbenzothiazoline-6-sulfonic acid) diammonium salt; FRAP: Ferric Reducing Antioxidant Power; AAPH: 2,2-azobis(2-amidinopropane) dihydrochloride.

## Data Availability

The data generated during the current study are available from the corresponding author on reasonable request.

## References

[1] Xu J., Wang X., Su G., Yue J., Sun Y., Cao J., Zhao Y. (2018). The antioxidant and anti-hepatic fibrosis activities of acorns (*Quercus liaotungensis*) and their natural galloyl triterpenes. J. Funct. Foods.

[2] Berumen J., Baglieri J., Kisseleva T., Mekeel K. (2020). Liver fibrosis: Pathophysiology and clinical implications. Wiley Interdiscip. Rev. Syst. Biol. Med..

[3] Shi L., Zhang X., Liu X., Jiang Y., Deng Y., Liu J. (2021). Cranberry (*Vacinium macrocarpon*) phytochemicals inhibit hepatic stellate cell activation and liver fibrosis. Food Biosci..

[4] Yuan L., Kaplowitz N. (2013). Mechanisms of Drug-induced Liver Injury. Clin. Liver Dis..

[5] Novo E., Busletta C., Bonzo L., Povero D., Paternostro C., Mareschi K., Ferrero I., David E., Bertolani C., Caligiuri A. (2010). Intracellular reactive oxygen species are required for directional migration of resident and bone marrow-derived hepatic pro-fibrogenic cells. J. Hepatol..

[6] Reyes-Agüero J.A., Rivera J.R., Flores J. (2005). Variación morfológica de “Opuntia” (“Cactaceae”) en relación con su domesticación en la altiplanicie meridional de México. Intercienc. Rev. Cienc. Tecnol. Am..

[7] Castellanos-Santiago E., Yahia E.M. (2008). Identification and Quantification of Betalains from the Fruits of 10 Mexican Prickly Pear Cultivars by High-Performance Liquid Chromatography and Electrospray Ionization Mass Spectrometry. J. Agric. Food Chem..

[8] Patel S. (2012). Reviewing the prospect of Opuntia pears as low cost functional foods. Rev. Environ. Sci. Bio Technol..

[9] Patil K., Dagadkhair A. (2019). Physicochemical characteristics and antioxidant potential of Opuntia fruit: A review Kartika V Patil and Amol C Dagadkhair. Seeds.

[10] Ali R. (2014). Antioxidant and Anticancer Activities of Different Constituents Extracted from Egyptian Prickly Pear Cactus (*Opuntia Ficus*-*Indica*) Peel Faten, M. Abou-Elella and Rehab Farouk Mohammed Ali* Department of. Biochem. Anal. Biochem..

[11] De Wit M., du Toit A., Osthoff G., Hugo A. (2020). Antioxidant Content, Capacity and Retention in Fresh and Processed Cactus Pear (*Opuntia ficus-indica* and *O. robusta*) Fruit Peels from Different Fruit-Colored Cultivars. Front. Sustain. Food Syst..

[12] González-Ponce H.A., Martínez-Saldaña M.C., Rincón-Sánchez A.R., Sumaya-Martínez M.T., Buist-Homan M., Faber K.N., Moshage H., Jaramillo-Juárez F. (2016). Hepatoprotective Effect of *Opuntia robusta* and *Opuntia streptacantha* Fruits against Acetaminophen-Induced Acute Liver Damage. Nutrients.

[13] Bankova V. (2005). Chemical diversity of propolis and the problem of standardization. J. Ethnopharmacol..

[14] Jiménez-Aguilar D.M., López-Martínez J.M., Hernández-Brenes C., Gutiérrez-Uribe J.A., Welti-Chanes J. (2015). Dietary fiber, phytochemical composition and antioxidant activity of Mexican commercial varieties of cactus pear. J. Food Compos. Anal..

[15] Chavez-Santoscoy A., Gutiérrez-Uribe J., Serna-Saldivar S. (2009). Phenolic Composition, Antioxidant Capacity and In Vitro Cancer Cell Cytotoxicity of Nine Prickly Pear (*Opuntia* spp.) Juices. Plant Foods Hum. Nutr..

[16] Chang S.F., Hsieh C.L., Yen G.C. (2008). The protective effect of *Opuntia dillenii* Haw fruit against low-density lipoprotein peroxidation and its active compounds. Food Chem..

[17] Budinsky A., Wolfram R., Oguogho A., Efthimiou Y., Stamatopoulos Y., Sinzinger H. (2001). Regular ingestion of opuntia robusta lowers oxidation injury. Prostaglandins Leukot. Essent. Fat. Acids (PLEFA).

[18] González-Ponce H.A., Martínez-Saldaña M.C., Tepper P.G., Quax W.J., Buist-Homan M., Faber K.N., Moshage H. (2020). Betacyanins, major components in *Opuntia* red-purple fruits, protect against acetaminophen-induced acute liver failure. Food Res. Int..

[19] Villa-Jaimes G.S., Aguilar-Mora F.A., González-Ponce H.A., Avelar-González F.J., Martínez Saldaña M.C.M., Buist-Homan M., Moshage H. (2022). Biocomponents from *Opuntia robusta* and *Opuntia streptacantha* fruits protect against diclofenac-induced acute liver damage in vivo and in vitro. J. Funct. Foods.

[20] Piga A. (2004). Cactus Pear: A Fruit of nutraceutical and functional importance. J. Prof. Assoc. Cactus Dev..

[21] AOAC. AOAC International (2000). Official Method 920.151 Solids (Total) in Fruits and Fruit Products.

[22] Torres-Bojórquez A.E., García O., Miranda-Lopez R., Anaberta C.M. (2017). Evaluation of antioxidant capacity, physicochemical characteristics and sensory profile of *Opuntia robusta* and *O. ficus-indica*. Arch. Latinoam. Nutr..

[23] Chaouch M., Hafsa J., Rihouey C., le Cerf D., Majdoub H. (2016). Effect of extraction conditions on the antioxidant and antiglycation capacity of carbohydrates from *Opuntia robusta* cladodes. Int. J. Food Sci. Technol..

[24] Van Soest P.J. (1994). Nutritional Ecology of the Ruminant.

[25] González Ponce H., Rincón-Sánchez A., Jaramillo-Juárez F., Moshage H. (2018). Natural Dietary Pigments: Potential Mediators against Hepatic Damage Induced by Over-The-Counter Non-Steroidal Anti-Inflammatory and Analgesic Drugs. Nutrients.

[26] Buettner G. (1993). The pecking order of free radicals and antioxidants: Lipid peroxidation, a -tocopherol, and ascorbate. Arch. Biochem. Biophys..

[27] Livrea M.A., Tesoriere L., Neelwarne B. (2012). Lipoperoxyl Radical Scavenging and Antioxidative Effects of Red Beet Pigments BT—Red Beet Biotechnology. Food and Pharmaceutical Applications.

[28] Krajka-Kuźniak V., Paluszczak J., Szaefer H., Baer-Dubowska W. (2013). Betanin, a beetroot component, induces nuclear factor erythroid-2-related factor 2-mediated expression of detoxifying/antioxidant enzymes in human liver cell lines. Br. J. Nutr..

[29] Marañón-Ruiz V.F., Rizo de la Torre L del C., Chiu-Zarate R. (2011). Caracterización de las propiedades ópticas de Betacianinas y Betaxantinas por espectroscopía Uv-Vis y barrido en Z. Superf. Vacío.

[30] Fernández-López J.A., Almela L., Obón J.M., Castellar R. (2010). Determination of Antioxidant Constituents in Cactus Pear Fruits. Plant Foods Hum. Nutr..

[31] Macheix J.J., Fleuriet A., Billot J. (1990). Fruit Phenolics.

[32] Galati E.M., Tripodo M.M., Trovato A., Miceli N., Monforte M.T. (2002). Biological effect of *Opuntia ficus indica* (L.) Mill. (Cactaceae) waste matter: Note I: Diuretic activity. J. Ethnopharmacol..

[33] Cazzola R., Cestaro B. (2011). Red wine polyphenols protect n−3 more than n−6 polyunsaturated fatty acid from lipid peroxidation. Food Res. Int..

[34] Hwang Y.P., Choi J.H., Han E.H., Kim H.G., Wee J.-H., Jung K.O., Jung K.H., Kwon K.-I., Jeong T.C., Chung Y.C. (2011). Purple sweet potato anthocyanins attenuate hepatic lipid accumulation through activating adenosine monophosphate–activated protein kinase in human HepG2 cells and obese mice. Nutr. Res..

[35] Toufektsian M.C., Salen P., Laporte F., Tonelli C., de Lorgeril M. (2011). Dietary Flavonoids Increase Plasma Very Long-Chain (n-3) Fatty Acids in Rats. J. Nutr..

[36] Villasante A., Patro B., Chew B., Becerra M., Wacyk J., Overturf K., Powell M.S., Hardy R.W. (2015). Dietary Intake of Purple Corn Extract Reduces Fat Body Content and Improves Antioxidant Capacity and n-3 Polyunsaturated Fatty Acid Profile in Plasma of Rainbow Trout, *Oncorhynchus mykiss*. J. World Aquac. Soc..

[37] Pérez-Torres I., Zúñiga Muñoz A., Beltrán-Rodríguez U., Díaz-Díaz E., Martínez-Memije R., Guarner Lans V. (2014). Modification of the liver fatty acids by *Hibiscus sabdariffa* Linnaeus (Malvaceae) infusion, its possible effect on vascular reactivity in a metabolic syndrome model. Clin. Exp. Hypertens..

[38] Miler M., Živanović J., Ajdžanović V., Oreščanin-Dušić Z., Milenković D., Konić-Ristić A., Blagojević D., Milošević V., Šošić-Jurjević B. (2016). Citrus flavanones naringenin and hesperetin improve antioxidant status and membrane lipid compositions in the liver of old-aged Wistar rats. Exp. Gerontol..

[39] Vargas Mendoza N. (2012). Efecto Hepatoprotector y Antioxidante del Extracto y los Principios Activos de Geranium shiedeanum. Ph.D. Thesis.

[40] Esatbeyoglu T., Wagner A.E., Motafakkerazad R., Nakajima Y., Matsugo S., Rimbach G. (2014). Free radical scavenging and antioxidant activity of betanin: Electron spin resonance spectroscopy studies and studies in cultured cells. Food Chem. Toxicol..

[41] Sumaya-Martínez M.T., Cruz-Jaime S., Madrigal-Santillán E., García-Paredes J.D., Cariño-Cortés R., Cruz-Cansino N., Valadez-Vega C., Martinez-Cardenas L., Alanís-García E. (2011). Betalain, Acid ascorbic, phenolic contents and antioxidant properties of purple, red, yellow and white cactus pears. Int. J. Mol. Sci..

[42] Butera D., Tesoriere L., Di Gaudio F., Bongiorno A., Allegra M., Pintaudi A.M., Kohen R., Livrea M.A. (2002). Antioxidant Activities of Sicilian Prickly Pear (*Opuntia ficus indica*) Fruit Extracts and Reducing Properties of Its Betalains: Betanin and Indicaxanthin. J. Agric. Food Chem..

[43] Amjadi S., Mesgari Abbasi M., Shokouhi B., Ghorbani M., Hamishehkar H. (2019). Enhancement of therapeutic efficacy of betanin for diabetes treatment by liposomal nanocarriers. J. Funct. Foods.

[44] Prior R.L., Wu X., Schaich K. (2005). Standardized Methods for the Determination of Antioxidant Capacity and Phenolics in Foods and Dietary Supplements. J. Agric. Food Chem..

[45] Roginsky V., Lissi E.A. (2005). Review of methods to determine chain-breaking antioxidant activity in food. Food Chem..

[46] Ou B., Huang D., Hampsch-Woodill M., Flanagan J.A., Deemer E.K. (2002). Analysis of Antioxidant Activities of Common Vegetables Employing Oxygen Radical Absorbance Capacity (ORAC) and Ferric Reducing Antioxidant Power (FRAP) Assays: A Comparative Study. J. Agric. Food Chem..

[47] Chisté R., Freitas M., Mercadante A., Fernandes E. (2014). Carotenoids are Effective Inhibitors of in vitro Hemolysis of Human Erythrocytes, as Determined by a Practical and Optimized Cellular Antioxidant Assay. J. Food Sci..

[48] Kanner J., Harel S., Granit R. (2001). BetalainsA New Class of Dietary Cationized Antioxidants. J. Agric. Food Chem..

[49] Sakihama Y., Maeda M., Hashimoto M., Tahara S., Hashidoko Y. (2011). Beetroot betalain inhibits peroxynitrite-mediated tyrosine nitration and DNA strand damage. Free. Radic. Res..

[50] Keser S., Celik S., Türkoğlu S., Yilmaz O., Turkoglu I. (2012). Hydrogen Peroxide Radical Scavenging and Total Antioxidant Activity of Hawthorn. Chem. J..

[51] Valko M., Leibfritz D., Moncol J., Cronin M.T.D., Mazur M., Telser J. (2007). Free radicals and antioxidants in normal physiological functions and human disease. Int. J. Biochem. Cell Biol..

[52] Musyarofah N., Susanto S., Aziz S.A., Suketi K., Dadang D. (2020). The diversity of ‘kristal’ guava (*Psidium guajava*) fruit quality in response to different altitudes and cultural practices. Biodiversitas.

[53] Alkaladi A. (2018). Vitamins E and C ameliorate the oxidative stresses induced by Zinc oxide nanoparticles on liver and gills of Oreochromis niloticus. Saudi J. Biol. Sci..

[54] El-Aal A.A., El-Ghffar E.A.A., Ghali A.A., Zughbur M.R., Sirdah M.M. (2018). The effect of vitamin C and/or E supplementations on type 2 diabetic adult males under metformin treatment: A single-blinded randomized controlled clinical trial. Diabetes Metab. Syndr. Clin. Res. Rev..

[55] Sumida Y., Niki E., Naito Y., Yoshikawa T. (2013). Involvement of free radicals and oxidative stress in NAFLD/NASH. Free Radic. Res..

[56] Al-Mehdar A.A., El-Denshary E.S., Addel-wahhab M.A. (2012). Alpha Lipoic Acid and Alpha-Tocopherol Counteract the Oxidative Stress and Liver Damage in Rats Sub-Chronically Treated with Khat (*Catha edulis*) Extract. Glob. J. Pharmacol..

[57] Abhilash P.A., Harikrishnan R., Indira M. (2014). Ascorbic acid suppresses endotoxemia and NF-κB signaling cascade in alcoholic liver fibrosis in guinea pigs: A mechanistic approach. Toxicol. Appl. Pharmacol..

[58] Gliszczyńska-Świgło A., Szymusiak H., Malinowska P. (2006). Betanin, the main pigment of red beet: Molecular origin of its exceptionally high free radical-scavenging activity. Food Addit. Contam..

[59] Tada M., Kohno M., Niwano Y. (2010). Scavenging or Quenching Effect of Melanin on Superoxide Anion and Singlet Oxygen. J. Clin. Biochem. Nutr..

[60] Brahmi D., Bouaziz C., Ayed Y., ben Mansour H., Zourgui L., Bacha H. (2011). Chemopreventive effect of cactus *Opuntia ficus indica* on oxidative stress and genotoxicity of aflatoxin B1. Nutr. Metab..

[61] Xie Y., Wang G., Wang H., Yao X., Jiang S., Kang A., Zhou F., Xie T., Hao H. (2012). Cytochrome P450 Dysregulations in Thioacetamide-Induced Liver Cirrhosis in Rats and the Counteracting Effects of Hepatoprotective Agents. Drug Metab. Dispos..

[62] Ramos-Tovar E., Casas-Grajales S., Hernández-Aquino E., Flores-Beltrán R.E., Galindo-Gómez S., Vera-Aguilar E., Diaz-Ruiz A., Montes S., Camacho J., Tsutsumi V. (2019). Cirrhosis induced by thioacetamide is prevented by stevia. Molecular mechanisms. J. Funct. Foods.

[63] El-Latif El-Ghazaly M.A., Rashed E.R., Shafey G.M., Zaki H.F., Attia A.S. (2020). Amelioration of thioacetamide-induced hepatic encephalopathy in rats by low-dose gamma irradiation. Environ. Sci. Pollut. Res..

[64] Gulbahar O., Karasu Z., Ersoz G., Akarca U., Musoglu A. (2000). Treatment of nonalcoholic steatohepatitis with N-acetylcysteine (Abstract). Gastroenterology.

[65] Bashandy S.A.E., el Awdan S.A., Mohamed S.M., Omara E.A.A. (2020). Allium porrum and Bauhinia Variegata Mitigate Acute Liver Failure and Nephrotoxicity Induced by Thioacetamide in Male Rats. Indian J. Clin. Biochem..

[66] Abul H., Mathew T.C., Dashti H.M., Al-Bader A. (2002). Level of Superoxide Dismutase, Glutathione Peroxidase and Uric Acid in Thioacetamide-Induced Cirrhotic Rats. Anat. Histol. Embryol..

[67] Tesoriere L., Allegra M., Gentile C., Livrea M. (2009). Betacyanins as phenol antioxidants. Chemistry and mechanistic aspects of the lipoperoxyl radical-scavenging activity in solution and liposomes. Free Radic. Res..

[68] Dwivedi D., Jena G. (2018). Glibenclamide protects against thioacetamide-induced hepatic damage in Wistar rat: Investigation on NLRP3, MMP-2, and stellate cell activation. Naunyn-Schmiedeberg’s Arch. Pharmacol..

[69] Yormaz S., Bulbuloglu E., Kurutas E.B., Ciralik H., Yuzbasioglu M.F., Yildiz H., Coskuner I., Silay E., Kantarceken B., Goksu M. (2012). The comparison of the effects of hepatic regeneration after partial hepatectomy, silybum marinaum, propofol, N-acetylcysteine and vitamin E on liver. Bratisl. Lekárske Listy.

[70] AOAC. AOAC International (1977). Official Method 954.02, Fat Crude or Ether Extract in Pet Food.

[71] Takoudjou Miafo A.P., Koubala B.B., Muralikrishna G., Kansci G., Fokou E. (2022). Non-starch polysaccharides derived from sorghum grains, bran, spent grain and evaluation of their antioxidant properties with respect to their bound phenolic acids. Bioact. Carbohydr. Dietary Fibre.

[72] Ilyas M., Khan W.A., Ali T., Ahmad N., Khan Z., Fazal H., Zaman N., Ualiyeva D., Ali M., Amissah O.B. (2022). Cold Stress-induced Seed Germination and Biosynthesis of Polyphenolics Content in Medicinally Important Brassica rapa. Phytomedicine Plus.

[73] Lasano N.F., Hamid A.H., Karim R., Dek M.S.P., Shukri R., Shazini Ramli N. (2019). Nutritional Composition, Anti-Diabetic Properties and Identification of Active Compounds Using UHPLC-ESI-Orbitrap-MS/MS in *Mangifera odorata* L.. Peel Seed Kernel. Mol..

[74] Ruslan K., Happyniar S., Fidrianny I. (2018). Antioxidant potential of two varieties of *Sesamum indicum* L. collected from Indonesia. J. Taibah Univ. Med. Sci..

[75] Xu P., Qian Y., Wang R., Chen Z., Wang T. (2022). Entrapping curcumin in the hydrophobic reservoir of rice proteins toward stable antioxidant nanoparticles. Food Chem..

[76] Khudyakov D., Sosnin M., Shorstkii I., Okpala C.O.R. (2022). Cold filamentary microplasma pretreatment combined with infrared dryer: Effects on drying efficiency and quality attributes of apple slices. J. Food Eng..

[77] Vinjamuri S., Shanker D., Ramesh R.S., Nagarajan S. (2015). In vitro evaluation of hemolytic activity and cell viability assay of hexanoic extracts of *Bridelia ferruginea* Benth. World J. Pharm. Pharm. Sci. (WJPPS).

[78] Khan S., Rehman M., Muhammed K., Khan M., Haq I., Khan M. (2022). In vitro and in vivo antioxidant therapeutic evaluation of *Dodonaea viscosa*. Biorxiv.

[79] Singh G., Mohanty B.P., Saini G.S.S. (2016). Structure, spectra and antioxidant action of ascorbic acid studied by density functional theory, Raman spectroscopic and nuclear magnetic resonance techniques. Spectrochim. Acta Part A Mol. Biomol. Spectrosc..

[80] Limbach J.R., Espinosa C.D., Perez-Calvo E., Stein H.H. (2021). Effect of dietary crude protein level on growth performance, blood characteristics, and indicators of intestinal health in weanling pigs. J. Anim. Sci..

[81] El-Baz F.K., Salama A.A.A., Hussein R.A. (2020). Dunaliella salina microalgae oppose thioacetamide-induced hepatic fibrosis in rats. Toxicol. Rep..

[82] Han J., Ma D., Zhang M., Yang X., Tan D. (2015). Natural Antioxidant Betanin Protects Rats from Paraquat-Induced Acute Lung Injury Interstitial Pneumonia. BioMed Res. Int..

[83] Prophet E.B., Prophet Edna (1992). Methods in Histotechnology.

